# Mitochondrial Dysfunction, Altered Mitochondrial Oxygen, and Energy Metabolism Associated with the Pathogenesis of Schizophrenia

**DOI:** 10.3390/ijms24097991

**Published:** 2023-04-28

**Authors:** Iveta Fizíková, Jozef Dragašek, Peter Račay

**Affiliations:** 1 Outpatient Psychiatry Clinic, 965 01 Žiar nad Hronom, Slovakia; yvett.fizikova@gmail.com; 2 1st Department of Psychiatry, Faculty of Medicine, University of P. J. Šafárik, 040 11 Košice, Slovakia; 3 Institute of Medical Biochemistry, Jessenius Faculty of Medicine, Comenius University, 036 01 Martin, Slovakia; racay@jfmed.uniba.sk

**Keywords:** schizophrenia, mitochondria, oxidative stress, energy metabolism

## Abstract

The significant complexity of the brain can lead to the development of serious neuropsychiatric disorders, including schizophrenia. A number of mechanisms are involved in the etiopathogenesis of schizophrenia, pointing to its complexity and opening a new perspective on studying this disorder. In this review of currently published studies, we focused on the contribution of mitochondria to the process, with an emphasis on oxidative damage, ROS, and energy metabolism. In addition, we point out the influence of redox imbalance, which can lead to the occurrence of oxidative stress with increased lipid peroxidation, linked to the formation of toxic aldehydes such as 4-hydroxynonenal (4-HNE) and HNE protein adducts. We also analysed the role of lactate in the process of energy metabolism and cognitive functions in schizophrenia.

## 1. Introduction

Schizophrenia is a specifically human disease that affects approximately 0.5–1% of the population. Schizophrenia has been defined as a neuropsychiatric disease that causes changes in the process of thoughts, perceptions, and emotions, usually leading to mental deterioration and affective blunting [1,2].

Currently, commonly used clinical treatments can improve some symptoms but not others; for example, negative symptoms such as social withdrawal and alogia in schizophrenia are not sufficiently treated with antipsychotic medications. In addition, it is currently not possible to predict which patients will or will not respond to antipsychotic medications. Therefore, developing novel, more effective therapeutic strategies for schizophrenia is imperative [2,3,4,5].

Early detection and intervention in schizophrenia requires mechanism-based biomarkers that capture circuitry dysfunction and can allow optimised patient stratification, disease monitoring, treatment, and improved prognosis [3,4,6].

The hypothesis of mitochondrial dysfunction as a factor in the etiopathogenesis of schizophrenia is currently gaining more attention. This is the basis of so-called mitochondrial psychiatry. The basis of this hypothesis is that mitochondria are important not only as the main energy producer but also as a significant influencer of many important life processes [1,5,6,7].

## 2. Mitochondria

Mitochondria, with rare exceptions, are found in all eukaryotic cells. Amongst their many roles, mitochondria are crucial in energy production, iron homeostasis, and the biosynthesis of lipids, amino acids, and nucleic acids [1,7,8,9].

Mitochondria are integral to functions within the central nervous system because they produce most of the energy needed for membrane ATPases, the influx and efflux of neurotransmitters, and the formation of new neural circuits. Mitochondria exhibit an adaptive response to the fluctuating needs of their host cells to maintain bioenergetic and oxidative homeostasis. In large complex cells such as neurons, mitochondrial distribution plays a critical role in supplying needed energy [1,6,7,8]. Mitochondria are also a significant source of intracellular ROS and NO; therefore, oxidative and nitrosative stress may be a downstream effect of mitochondrial dysfunction. Mitochondria play an essential role in normal physiology and in various pathological conditions, including schizophrenia [1,7,9,10,11]. Various tissues have different susceptibilities to oxidative stress. The brain is particularly vulnerable to oxidative damage due to relatively low levels of antioxidants, high levels of polyunsaturated fatty acids, high metal content, and oxygen utilisation [10,11,12].

Oxidative stress has become an attractive hypothesis to explain the pathophysiology of schizophrenia based on changes caused by free radicals and dysregulation of the antioxidant defence system. Oxidative and nitrosative damage to nucleic acids, proteins, and lipids impairs the viability, survival, and function of neurons and glial cells, which induces or accompanies processes associated with the onset and progression of schizophrenia, such as impaired neurodevelopment, neurotransmission, and intracellular and intercellular signalling, immune dysfunction, and neuroinflammation [1,12,13].

Ample evidence shows that mitochondria are involved not only in early brain development but also in the late brain development that occurs throughout adolescence, a critical period in the pathogenesis of various psychiatric disorders, specifically schizophrenia [13]. Under conditions of chronic oxidative stress, there is an increase in the levels of oxidised proteins, which can lead to cell dysfunction and cell death. Postmortem studies provide evidence of regional reductions in neuronal and glial density, suggesting that apoptosis may be involved in the development of schizophrenia. Apoptotic activity at the onset of schizophrenia is thought to contribute to reduced neuronal survival and the disruption of synaptic plasticity. In response to intracellular signals generated by cellular stress and mitochondrial dysfunction, the intrinsic apoptosis pathway can be triggered by the release of proapoptotic factors from mitochondria [8,12,13,14].

## 3. Markers of Oxidative Stress in Schizophrenia

Fluorescence analysis is a simple, fast, and relatively reliable way to determine the level of oxidative stress in vivo. Changes in the fluorescence of tryptophans, dityrosines, and lysine conjugates with lipid peroxidation products of proteins from various sources are considered important indicators. Tryptophan is an amino acid that is susceptible to oxidation, and reacts with various types of ROS under physiological conditions. In most cases, a decrease in tryptophan fluorescence can provide helpful information about changes in the structure of tryptophan residues caused by ROS. It can therefore be considered an important oxidative stress marker [15].

For example, the significantly decreased fluorescence in tryptophan residues of membrane proteins after incubating endoplasmic reticulum membranes with Fe^2+^ as a source of superoxide anion radicals has been documented [16,17].

Several studies have investigated schizophrenia-associated plasma protein modifications induced by oxidative stress using different methods. For instance, statistically increased markers of oxidative/nitrative stress, such as carbonyl groups or 3-nitrotyrosine, as determined by ELISA, have been observed in the plasma protein of patients with schizophrenia [18,19].

Another study documented that spectrophotometrically determined plasma protein oxidation levels were significantly higher in patients during an acute psychotic episode of schizophrenia compared to a control group. That study also demonstrated that, although the level of protein oxidation did not change significantly in schizophrenia patients in remission compared to the control group, the level of total oxidised guanine was statistically higher in patients during an acute psychotic episode and those in remission compared to the control group [20].

Our recent study documented a significant decrease in the fluorescence of tryptophan in the plasma protein of patients suffering from schizophrenia. In contrast, other investigated parameters (fluorescence corresponding to the modification of plasma proteins with HNE and formation of dityrosines) did not significantly change (Table 1) [21].

Protein carbonylation, a form of protein modification caused by oxidative stress, is also a suitable marker of oxidative stress due to its irreversibility and relevant stability. Oxidative carbonylation is a non-enzymatic phenomenon that leads to protein dysfunction based on the direct or secondary reaction of oxidants with the given protein. The direct reaction refers to the metal-catalysed ROS attack on amino acids (e.g., proline, arginine, lysine, or threonine). This reaction produces more products than glutamate semialdehyde for proline or aminoadipic semialdehyde for lysine. In addition, carbonylated side chains increase the overall hydrophobicity of proteins due to their unfolding, leading to an increased risk of aggregation [22,23].

Furthermore, secondary reactions generating carbonyls on proteins can occur due to oxidative stress. Protein carbonylation can also occur as a result of the reaction of modified aldehydes (lipid peroxidation products), such as 4-hydroxynonenal (HNE) with specific amino acids [22,23,24].

The study of biological markers and their importance in schizophrenia is becoming essential, opening up a new perspective on schizophrenia (Table 2).

## 4. The Link between HNE and Schizophrenia

HNE is an essential molecule that significantly impacts cell function and survival. For this reason, it is assumed that HNE might play a role in the pathophysiology of schizophrenia and other disorders (e.g., Rett syndrome and autism spectrum disorder), although its specific influence is not yet fully understood [24,26,27,28,29]. Regarding schizophrenia, the results of a recent study showed increased levels of HNE conjugates with proteins in the brains of patients with schizophrenia compared to the control group. Interestingly, elevated levels of HNE protein conjugates have been documented in the hippocampus compared to the cortex of schizophrenia patients [24].

**Figure 1:**
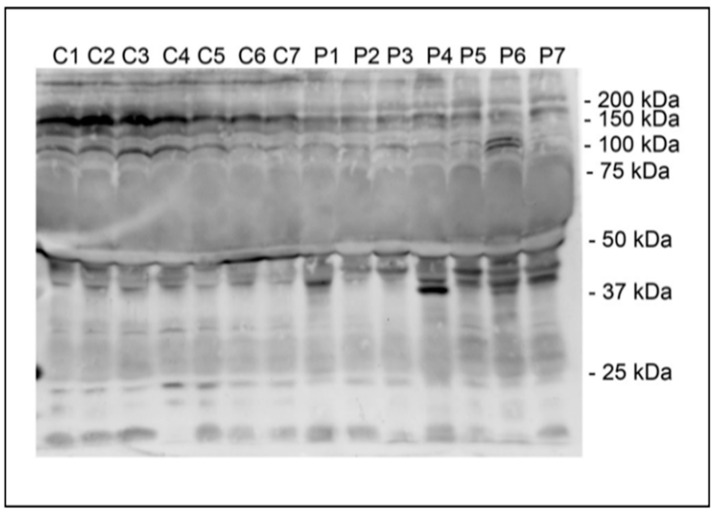
Representative Western blot detection of HNE conjugates of plasma proteins of control volunteers (C) and schizophrenia patients (P). Positions of molecular mass standards are indicated. Adapted from [21].

In our recent study [21], Western blot analysis showed HNE-modified plasma proteins with a discrete molecular mass in the range of 37–50 kDa in the plasma of schizophrenia patients, while HNE-modified plasma proteins with a discrete molecular mass of 100–200 kDa were documented in the control group (Figure 1).

The altered profile of plasma proteins modified by HNE may explain the statistically insignificant results obtained by fluorescence measurements [21].

Interestingly, in one study, increased levels of HNE-modified proteins with a molecular weight of around 50 kDa were observed in the plasma of patients suffering from Rett syndrome [27]. That study also documented increased levels of HNE-modified proteins with a molecular weight of 150–200 kDa. The massive modification of HNE plasma proteins in the plasma of patients with classical autistic disorder was also reported [28,29].

In another study, an increase in discrete proteins modified by HNE was observed, mainly in mitochondria isolated from rat brains, and was dependent on age [29].

It is also known that free HNE can react with proteins, thereby changing their conformation and function, as was also shown in the case of cytochrome c oxidase, the terminal complex of the mitochondrial respiratory chain. As a result of the action of HNE, various intracellular enzymes can also be inhibited (e.g., Na/K-ATPase, poly-ADP ribose polymerase, complex I of the respiratory chain, protein kinase C, NADPH oxidase, and proteasome). The inhibitory values reported in the literature indicate a wide range of enzyme sensitivity to HNE. Although HNE is primarily generated in membranes (or other hydrophobic compartments of cells), the partition coefficient of HNE allows diffusion into the cytosol or the extracellular space [23,24].

Nevertheless, it can be assumed that the HNE concentration is much higher in cell membranes than in the hydrophilic compartments of cells. HNE is considered a bifunctional aldehyde and can easily form cross-links within or between proteins. HNE is formed under physiological conditions and in pathophysiological situations as a product of ongoing oxidative stress; therefore, it is likely that many proteins are directly damaged by oxidants. The groups of HNE-peptide and HNE-protein conjugates are an integral part of the harmful effects of HNE on cellular functions [11,23,30,31].

In contrast, other pathways of HNE degradation contribute to HNE detoxification. These pathways include products of beta- and alpha-oxidation and the tricarboxylic acid cycle, such as acetyl-CoA, citrate, aconitate, malate, fumarate, succinate, carbon dioxide, and water. The most critical HNE-degrading enzymes are glutathione-S transferase, alcohol dehydrogenase, and aldehyde dehydrogenase. Among the products generated by these enzymatic substances, referred to as primary intermediates of HNE, are HNE-GSH, hydroxynonenic acid (HNA), and 1,4-dihydroxynonene (DH) [11,30,31].

The quick elimination of HNE from affected cells is a significant part of the secondary antioxidant defence mechanism of cells. Still, the processes have not yet been studied in detail with regard to schizophrenia. In addition, the increased formation of protein conjugates with HNE may also be a consequence of the activation of the inflammatory response, which is often involved in the etiopathogenesis of schizophrenia [11,30,32,33,34].

As mentioned, one of the specific targets of HNE may be cytochrome c oxidase, the terminal complex of the mitochondrial respiratory chain [32]. Modifying cytochrome c oxidase via HNE inhibits its activity, which could lead to mitochondrial dysfunction and lactate overproduction. Changes in lactate levels may be evidence of impaired energy metabolism in schizophrenia [34,35].

## 5. Changes in Energy Metabolism in Schizophrenia

The disruption of brain cell function, neuroplasticity, and brain circuits in schizophrenia can be caused by impaired energy metabolism [5,6,36,37] and increased oxidative stress, which are processes regulated primarily by mitochondria. In addition, there is evidence to suggest that mitochondrial dysfunction and oxidative stress participate in the pathophysiology of schizophrenia [6,10,37].

Bioenergetic requirements in the brain require the coordination of several systems and cell types for the sufficient provision of energy substrates. To meet the energy demands of neurons, the brain mainly uses glycolysis, oxidative phosphorylation, and lactate absorption [35,36].

Glycolysis and oxidative metabolism via the tricarboxylic acid (TCA) cycle and oxidative phosphorylation are key pathways in maintaining synaptic function. Neurons and astrocytes use glycolysis even under aerobic conditions. Glucose, which is involved in the glycolytic pathway, enters the cell via glucose transporters (GLUTs) or arises from the breakdown of glycogen in astrocytes. Meeting the energy requirements of neurons significantly depends on the metabolic cooperation of neurons with glycolysis and lactate production in astrocytes. Key enzymes in glycolysis include hexokinase (HXK) and lactate dehydrogenase (LDH). Hexokinase is the initial enzyme of glycolysis as it catalyses the phosphorylation of glucose by ATP to glucose-6-P. It is one of the rate-limiting enzymes of glycolysis. LDH catalyses the conversion of lactate to pyruvate and back, as it converts NAD^+^ to NADH and back [1,36,37].

This metabolic pathway also requires the presence of monocarboxylate transporters (MCTs), which enable the rapid transport of lactate generated in astrocytes into the extracellular space and neurons.

At this point, lactate is converted to pyruvate by LDH, which can enter the TCA cycle and oxidative phosphorylation to form adenosine triphosphate (ATP) molecules. The flow of energy from astrocytes to neurons that ensures neuronal activity is called the astrocyte–neuron–lactate shuttle. Brain activity and function are highly dependent on the supply of ATP, with energy requirements varying significantly depending on neuron type and brain activity level. Creatine is phosphorylated and converted by creatine kinase into phosphocreatine (PCr), which is a highly energetic phosphate capable of transferring the phosphate group of adenosine diphosphate (ADP) to form ATP and vice versa [36].

In patients with schizophrenia, imaging studies using positron emission tomography (PET), functional magnetic resonance imaging (fMRI), magnetic resonance spectroscopy (MRS), phosphor magnetic resonance spectroscopy (P-MRS), and single-photon emission tomography (SPECT) have revealed altered metabolisms, as expressed by changes in glucose, PCr, and ATP in various brain regions, including the prefrontal cortex (PFC), left temporal lobe, and frontal lobe [36,37].

Interestingly, in patients with schizophrenia, the severity of negative symptoms and the neuropsychological manifestations have been correlated with ATP and PCr levels. Conversely, in studies of patients with bipolar disorder (BD) who also had psychotic symptoms and shared a genetic risk for schizophrenia, P-MRS did not confirm significant changes in ATP [38,39]. However, similar changes were noted in both disorders in other bioenergetic indicators, such as increased lactate, decreased intracellular pH, and abnormalities in PCr and creatine kinase. Therefore, it is hypothesised that switching from aerobic respiration to anaerobic glycolysis increases the risk of metabolic syndromes in these patients [38,39,40].

Oxidative phosphorylation (OXPHOS) dysfunction in schizophrenia has been observed at the high-energy phosphate production level and in enzymatic activity and the expression of subunits of OXPHOS complexes. About 90% of the ATP molecules created by OXPHOS in the brain provide energy for cellular signalling and processes of neuronal activity, such as pre- and postsynaptic action potential and neurotransmitter release. Therefore, the disruption of OXPHOS, has an adverse effect on the energy balance of the CNS, which can lead to various neuronal dysfunctions [38,41,42,43].

Many studies have found defects in various components of the OXPHOS protein apparatus in schizophrenia. The ability of OXPHOS to interact with antipsychotics and dopamine suggests that mitochondria, in general, and CoI, in particular, could serve as new targets for future therapeutic strategies [42,43].

The mitochondrial DNA (mtDNA) content is a quantifiable biomarker of mitochondrial status because it directly correlates with mitochondrial biogenesis, transcription, and translation. A low mtDNA level leads to decreased OXPHOS and ATP levels, which can lead to mitochondrial dysfunction. mtDNA levels directly correlate with energy reserves, oxidative stress, and changes in mitochondrial membrane potential [44]. The decreased levels of mtDNA documented in leukocytes of schizophrenia patients [21] might indirectly indicate mitochondrial dysfunction, which is often included in the pathophysiologic mechanisms of schizophrenia [44,45].

Several studies have demonstrated that the reduced mtDNA copy number in the leukocytes of schizophrenia patients is independent of antipsychotic treatment. In contrast, other studies have shown that the mtDNA copy number in the leukocytes of schizophrenia patients is associated with psychosis severity and antipsychotic treatment [44]. In addition, the reduced mtDNA copy numbers in the leukocytes of patients with bipolar disorder, which may reflect disturbances in energy metabolism, has also been documented [39]. Conversely, higher mtDNA copy numbers in leukocytes have been reported in association with ADHD [46,47].

## 6. The Role of Lactate in the Process of Energy Metabolism

Lactate acts as a buffer between glycolysis and oxidative metabolism. In this process, it is exchanged as a substrate between cells and tissues with different glycolytic and oxidative requirements. According to several studies, lactate is the preferred substrate for brain energy metabolism [38,48,49,50].

At rest, there is an outflow of lactate from the brain into the blood. However, when the level of lactate in the blood increases, for example, after physical exercise, there is an influx of lactate from the blood to the brain [48].

Glucose is the primary source of energy for the adult brain. Cells can store glucose as glycogen or break it down into pyruvate during glycolysis. The rapid generation of ATP through glycolysis is essential after neuronal activation and could explain the immediate increase in glucose uptake when oxygen uptake lags. Likewise, there is an immediate decrease in lactate after neuronal activation, followed by a rise after a few seconds, indicating that the cells have a latent capacity to oxidise rapidly depleted lactate. The chemical energy of glucose is released as ATP by metabolising pyruvate in the mitochondria through the tricarboxylic acid cycle and OXPHOS [38,48].

When the rate of glycolysis exceeds the rate of oxidative phosphorylation, cells convert pyruvate to lactate using lactate dehydrogenase. Lactate can exit cells via monocarboxylate transporter (MTC) and be metabolised in other cells that also carry MTC. The co-transport of protons and lactate through the MTC affects pH regulation [48].

The monocarboxylate transporter consists of several isoforms with different affinities for lactate and other substrates. The high-affinity isoforms MTC1 and MTC2 are expressed in cells that mainly import lactate. The low-affinity isoform MCT4 is predominantly expressed in cells that export lactate. MTC4 also has a very low affinity for pyruvate, thereby saving pyruvate from being converted into lactate via lactate dehydrogenase, while simultaneously converting NADH to NAD, which maintains glycolysis [49,50].

Brain-derived neurotrophic factor (BDNF) increases a level of MTC2, but not MTC1 and MTC4, through several signalling mechanisms involved in synaptic plasticity [51]. Norepinephrine, similar to insulin and insulin-like growth factor, also causes the upregulation of MCT2 via mitogen-activated protein kinase and the mammalian target of rapamycin. The MTC4 isoform, characterised by its low affinity and high capacity, is found on astrocytes. We classify astrocytes as glycolytic cells that can supply lactate to other cells. Due to the high Km (around 30 mmol/L), MCT4 is usually not saturated; therefore, the transport rate increases with increasing lactate concentration. MTC2 is mainly found in cell bodies, dendrites, dendritic spines and axons, which are highly oxidative and probably particularly import lactate. A Km of less than 1 mmol/L indicates that MCT2 will be saturated with lactate in most cases, and that the rates of efflux and absorption will be largely independent of lactate concentration. MTC1 is mainly detected in the endothelial cells of the blood–brain barrier [48,49].

A possible neuroprotective effect of lactate in brain injuries is interesting. The process of neuroprotection consists of reoxygenation with possible use after stroke or trauma. At the same time, lactate reduces glutamate-induced neurotoxicity in the cerebral cortex in vivo. Lactate administration, with an increased systemic blood lactate of 5 mmol/L, has a beneficial effect in brain-injured patients with low lactate, glucose, pyruvate levels, and reduced intracranial pressure [48].

## 7. The Role of Lactate in Schizophrenia

Schizophrenia has a broad spectrum of psychotic symptoms, cognitive deficits, and severe negative symptoms. In schizophrenia patients, response to antipsychotic therapy is highly variable. Approximately 30% of schizophrenia patients are classified as treatment-resistant [2,5,6]. Cognitive functions are closely linked to synaptic activity, with the emphasis placed on the ability of cells to sufficiently generate bioenergetic substrates [48,52].

For example, the release of glutamate leads to increased energy demand by astrocytes and the production of bioenergetic substrates such as lactate. Lactate is then transported via MTC from astrocytes to neurons and is used for energy production in the tricarboxylic acid cycle.

This mechanism, referred to as the astrocyte–neuron–lactate shuttle, uses lactate to support neuronal oxidative phosphorylation even under aerobic conditions when energy demand is high and neurons are unable to regulate glycolysis efficiently [52].

An animal study of adult rats also confirmed the hypothesis that lactate is necessary for learning: an increase in extracellular lactate improved the performance of learning tasks, and vice versa, disruption of lactate production and its import into neurons led to amnesia. Lactate is essential for long-term memory and synaptic activity [48,52,53].

The results of several studies have demonstrated an impaired supply and transport of lactate in patients with schizophrenia. Elevated lactate concentrations were measured in cerebrospinal fluid (CSF) and stimulated peripheral blood mononuclear cells (PBMCs) obtained from schizophrenia patients. Studies with PBMCs have shown a significantly increased protein expression of lactate dehydrogenase B (LDHB) and glucose-6-phosphate isomerase (GPI) in patients with first-episode psychosis. Increased expression was observed in the treatment-naïve cohort and in the cohort of patients receiving antipsychotics for the first time [54].

Since LDH and GPI are glycolytic enzymes required for metabolising glucose into bioenergetic substrates, these findings support the hypothesis that the biological process involved in lactate production is abnormal in schizophrenia and likely contributes to the cognitive deficits confirmed in this disease [48,52].

Several MRS studies have demonstrated increased lactate levels in the brains of schizophrenia patients in vivo. This finding indicates a metabolic dysfunction in schizophrenia with a shift to anaerobic glycolysis. In the clinical picture of schizophrenia, patients with increased lactate levels as well as lower general cognitive function and functional capacity, including impairments in visual learning, processing speed, and cognitive domains of reasoning (problem-solving), have been reported [48,50,52].

Based on these results, several metabolomic analyses based on high-resolution ^1^H NMR spectroscopy (HRMAS) were performed, revealing a 1.5-fold increase in lactate in the white matter of the prefrontal cortex in schizophrenia [50]. Similarly, postmortem studies have shown increased lactate levels in the striatum and cerebellum of individuals with schizophrenia compared to controls [53].

Despite these findings, it is still not completely clear whether the lactate changes reported in postmortem studies are part of the pathophysiological mechanism related to the disease or are secondary changes due to the effects of drugs or other postmortem factors that can affect lactate levels [53].

## 8. Conclusions

Schizophrenia is an often debilitating illness that typically manifests in late adolescence and frequently has a progressive course associated with significant disability, economic burden, and early mortality. Therefore, an improved understanding of the molecular pathophysiology underlying schizophrenia is needed in order to better target future therapies.

**Figure 2:**
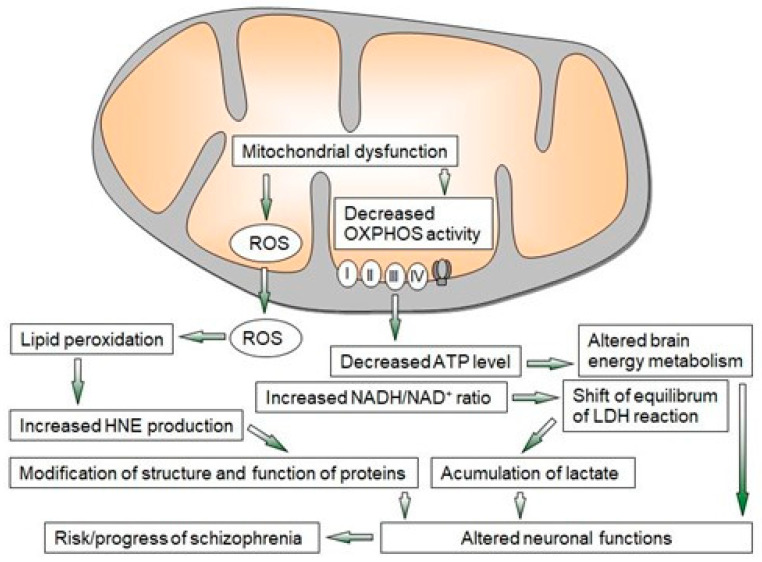
Overview of mechanisms of mitochondrial dysfunction leading to neuroprogressive changes in schizophrenia. LDH—lactate dehydrogenase, OXPHOS—oxidative phosphorylation, ROS—reactive oxygen species, I–IV—enzyme complexes of OXPHOS.

Multiple converging lines of evidence from ex vivo, postmortem, imaging, and animal model studies indicate that proper mitochondrial function is important in the pathogenesis of schizophrenia. The mitochondrial dysfunction observed in schizophrenia is closely related to oxidative stress with the formation of free radicals (Figure 2). These changes lead to oxidative damage and the alteration of several key enzymes and antioxidant systems, as well as energy metabolism [2,6,11,38,55,56].

In vitro measurement data suggest that the mitochondrial effects of current antipsychotics are likely related to their adverse impacts and are due to drug-induced decreased ATP production and increased ROS production; however, based on the mitochondrial dysfunctions observed in neurodegenerative diseases, bipolar disorder, and schizophrenia, new psychotropic drugs are aimed at selected mitochondrial targets [5,6].

Identifying the changes described above opens up new possibilities for therapeutic goals or the development of therapeutic procedures for schizophrenia, focusing on strengthening the antioxidant defence or reducing the consequences of oxidative damage.

Several studies have pointed to the effects of chemicals that increase mitochondrial function, which have been used as neoadjuvant treatments for schizophrenia. For example, omega-3 polyunsaturated fatty acids (PUFAs), including eicosapentaenoic acid (EPA) and docosahexaenoic acid (DHA), can reorganise the composition of mitochondrial membranes while promoting mitochondrial kinetics. Add-on treatment with PUFAs proved to be effective in ameliorating depression and anxiety symptoms and yielded significant improvements in the symptoms of schizophrenia [57,58,59,60].

N-acetyl cysteine (NAC) was shown to improve positive and negative symptoms in patients with schizophrenia and disorganised thought symptoms by activating the expression of mitochondria-related genes. Better auditory processing, cognitive function, and working memory performance have also been reported after NAC treatment in patients with schizophrenia. In addition, imaging analysis demonstrated that add-on treatment with NAC might contribute to protecting white matter integrity in patients with early psychosis [61,62].

Adjuvant treatment with alpha-lipoic acid/acetyl-L-carnitine (ALA/ALC) alongside antipsychotic medication has shown some promise in improving negative and cognitive symptoms in patients with schizophrenia. In addition, add-on treatment with CoQ10, an essential cofactor involved in the mitochondrial electron transport chain, was also found to benefit treatment outcomes [5].

These results suggest that there is potential for the use of mitochondrial function-enhancing agents as adjuvant treatments of psychiatric diseases in order to further optimise treatment for patients. In addition, using mitochondria-enhancing chemicals could also provide better control of the metabolic side effects of antipsychotics. However, large-scale clinical trials will be needed to convincingly determine their utility as a treatment for various psychiatric disorders.

## Figures and Tables

**Table 1:** Fluorescence intensity of modified amino acid residues of plasma proteins [21].

Fluorescence Intensity (a. u./mg of Protein)	Controls	Patients	*p* Value
Aromatic amino acids	20.41 ± 2.73	11.74 ± 2.64	0.0001
Dityrosine	2.95 ± 0.44	2.76 ± 0.81	0.34
HNE conjugates	6 ± 1.04	5.7 ± 1.75	0.47

**Table 2:** Biological markers of oxidative stress in schizophrenia.

Marker	Method	Material	Result	References
Carbonyl groups/3-nitro tyrosine	ELISA	Plasma	Elevated in schizophrenia	[18,19]
Protein oxidation	Spectroscopy	Plasma	Elevated in schizophrenia acute psychothic attack	[20]
Total oxidized guanine species	ELISA	Serum	Elevated in schizophrenia	[20]
Tryptophan	Fluorescence	Plasma	Decreased in schizophrenia	[21]
8-Hydroxy-2′-Deoxyguanosine	Flow cytometry ELISA Liquid chromatography with tandem mass spectrometry	Lymphocytes Plasma cell-free DNA Serum Plasma Urine	Elevated in different forms of schizophrenia	Summarised in [25]

## References

[1] Nestler E., Hyman E.S., Malenka R.C., Piško (2012). Molekulárna Neuropsychofarmakológia, Základy Klinických Neurovied.

[2] Vita A., Minelli A., Barlati S., Deste G., Giacopuzzi E., Valsecchi P., Turrina C., Gennarelli M. (2019). Treatment-Resistant Schizophrenia: Genetic and Neuroimaging Correlates. Front. Pharmacol..

[3] Fusar-Poli P., Papanastasiou E., Stahl D., Rocchetti M., Carpenter W., Shergill S., McGuire P. (2015). Treatments of Negative Symptoms in Schizophrenia: Meta-Analysis of 168 Randomized Placebo-Controlled Trials. Schizophr. Bull..

[4] Do K.Q. (2023). Bridging the gaps towards precision psychiatry: Mechanistic biomarkers for early detection and intervention. Psychiatry Res..

[5] Peiyan N. Mitochondrial dysfunction in psychiatric disorders. Schizophr. Res..

[6] Fišar Z. (2023). Biological hypotheses, risk factors, and biomarkers of schizophrenia. Prog. Neuropsychopharmacol. Biol. Psychiatry.

[7] Zilocchi M., Broderick K., Phanse S., Aly K.A., Babu M. (2020). Mitochondria under the spotlight: On the implications of mitochondrial dysfunction and its connectivity to neuropsychiatric disorders. Comput. Struct. Biotechnol. J..

[8] Vringer E., Tait S.W.G. (2023). Mitochondria and cell death-associated inflammation. Cell Death Differ..

[9] Monsour M., Gordon J., Lockard G., Alayli A., Borlongan C.V. (2023). Mitochondria in Cell-Based Therapy for Stroke. Antioxidants.

[10] Morris G., Walder K.R., Berk M., Marx W., Walker A.J., Maes M., Puri B.K. (2020). The interplay between oxidative stress and bioenergetic failure in neuropsychiatric illnesses: Can we explain it and can we treat it?. Mol. Biol. Rep..

[11] Ciobica A., Padurariu M., Dobrin M., Stefanescu C., Dobrin R. (2011). Oxidative stress in schizophrenia—Focusing on the main markers. Psychiatr. Danub..

[12] Murray A.J., Rogers J.C., Katshu M., Liddle P.F., Upthegrove R. (2021). Oxidative stress and the pathophysiology and symptom profile of schizophrenia spectrum disorders. Front. Psychiatry.

[13] Ene H.M., Karry R., Farfara D., Ben-Shachar D. (2023). Mitochondria play an essential role in the trajectory of adolescent neurodevelopment and behavior in adulthood: Evidence from a schizophrenia rat model. Mol. Psychiatry.

[14] Glantz L.A., Gilmore J.H., Lieberman J.A., Jarskog L.F. (2006). Apoptotic mechanismsand the synaptic pathology of schizophrenia. Schizophr. Res..

[15] Ehrenshaft M., Deterding L.J., Mason R.P. (2015). Tripping up Trp: Modification of protein tryptophan residues by reactive oxygen species, modes of detection, and biological consequences. Free Radic. Biol. Med..

[16] Kaplán P., Doval M., Majerová Z., Lehotský J., Racay P. (2000). Iron-induced lipid peroxidation and protein modification in endoplasmic reticulum membranes. Protection by stobadine. Int. J. Biochem. Cell Biol..

[17] Kaplán P., Babušíková E., Lehotský J., Dobrota D. (2003). Free radical-induced protein modification and inhibition of Ca^2+^-ATPase of cardiac sarcoplasmic reticulum. Mol. Cell. Biochem..

[18] Dietrich-Muszalka A., Malinovska J., Olas B., Głowacki R., Bald E., Wachowicz B., Rabe-Jabłońska J. (2012). The oxidative stress may be induced by the elevated homocysteine in schizophrenic patients. Neurochem. Res..

[19] Dietrich-Muszalka A., Malinovska J., Olas B., Bald E. (2009). Oxidative/nitrative modifications of plasma proteins and thiols from patients with schizophrenia. Neuropsychobiology.

[20] Tuncel O.K., Sansoy G., Bilgici B., Pazvantoglu O., Çetin E., Bahattin Avcı E.U., Böke O. (2015). Oxidative stress in bipolar and schizophrenia patients. Psychiatry Res..

[21] Fizikova I., Racay P. (2022). Oxidative modifications of plasma proteins and decreased leukocyte mitochondrial DNA of schizophrenia patients. Act. Nerv. Super. Rediviva.

[22] Martins-de-Souza D., Harris L.W., Guest P.C., Bahn S. (2011). The role of energy metabolism dysfunction and oxidative stress in schizophrenia revealed by proteomics. Antioxid. Redox Signal..

[23] Moller I.M., Rogowska-wrzesinska R.S., Rao R.S. (2011). Protein carbonylation and metal-catalysed Protein oxidation in a cellular perspective. J. Proteom..

[24] Manzoor S., Khan A., Hasan B., Mushtag S., Ahmed N. (2022). Expression Analysis of 4-hydroxynonenal Modified Proteins in Schizophrenia Brain; Relevance to Involvement in Redox Dysregulation. Curr. Proteom..

[25] Goh X.X., Tang P.Y., Tee S.F. (2021). 8-Hydroxy-2′-Deoxyguanosine and Reactive Oxygen Species as Biomarkers of Oxidative Stress in Mental Illnesses: A Meta-Analysis. Psychiatry Investig..

[26] Dalleau S., Baradat M., Guéraud F., Huc L. (2013). Cell death and diseases related to oxidative stress: 4-hydroxynonenal (HNE) in the balance. Cell Death Differ..

[27] Pecorelli A., Ciccoli L., Signorini C., Leoncini S., Giardini A., D’Esposito M., Filosa S., Hayek J., De Felice C., Valacchi G. (2011). Increased levels of 4HNE-protein plasma adducts in Rett syndrome. Clin. Biochem..

[28] Pecorelli A., Leoncini S., De Felice C., Signorini C., Cerrone C., Valacchi G., Ciccoli L., Hayek J. (2013). Non-protein-bound iron and 4-hydroxynonenal protein adducts in classic autism. Brain Dev..

[29] Pecorelli A., Leoncini C., Ciccoli S., Valacchi G., Signorini C., De Felice C., Hayek C. (2014). 4HNE Protein Adducts in Autistic Spectrum Disorders: Rett Syndrome and Autism. Comprehensive Guide to Autism.

[30] Tatarkova Z., Kovalska M., Timkova V., Racay P., Lehotsky J., Kaplan P. (2016). The Effect of Aging on Mitochondrial Complex I and the Extent of Oxidative Stress in the Rat Brain Cortex. Neurochem. Res..

[31] Siems W., Grune T. (2003). Intracellular metabolism of 4-hydroxynonenal. Mol. Asp. Med..

[32] Castro J.P., Jung T., Grune T., Siems W. (2017). 4-Hydroxynonenal (HNE) modified proteins in metabolic diseases. Free. Radic. Biol. Med..

[33] Feigenson K.A., Kusnecov A.W., Silverstein S.M. (2014). Inflammation and the two-hit hypothesis of schizophrenia. Neurosci. Biobehav. Rev..

[34] Fragaus D., Diaz-Caneja C.M., Ayora M., Hernández-Álvarez F., Rodríguez-Quiroga A., Recio S., Leza J.C., Arango C. (2019). Oxidative Stress and Inflammation in First-Episode Psychosis: A Systematic Review and Meta-analysis. Schizophr. Bull..

[35] Kaplán P., Tatarková Z., Račay P., Doborota D., Lehotsky J., Pavlikova M. (2007). Oxidative modifications of cardiac mitochondria and inhibition of cytochrome c oxidase activity by 4-hydroxynonenal. Redox Rep..

[36] Sullivan C.R., O’Donovan S.M., Mc Cullumsmith R.E., Ramsey A. (2018). Defects in Bioenergetic Coupling in Schizophrenia. Biol. Psychiatry.

[37] Duarte J.M.N., Xin L. (2019). Magnetic resonance spectroscopy in schizophrenia: Evidence for glutamatergic dysfunction and impaired energy metabolism. Neurochem. Res..

[38] Pruett B.S., Meador-Woodruff J.H. (2020). Evidence for altered energy metabolism, increased lactate, and decreased pH in schizophrenia brain: A focused review and meta-analysis of human postmortem and magnetic resonance spectroscopy studies. Schizophr. Res..

[39] Clay B., Sillivan S., Konradi C. (2011). Mitochondrial dysfunction and pathology in bipolar disorder and schizophrenia. Int. J. Dev. Neurosci..

[40] Fizikova I., Dragasek J. (2017). Mitochondrial dysfunction and new therapeutic targets in bipolar affective disorder. Psychiatr. Ann..

[41] Zuccoli G.S., Saia-Cereda V.M., Nascimento J.M., Martins-de-Souza D. (2017). The energy metabolism dysfunction in psychiatric disorders postmortem brains: Focus on proteomic evidence. Front. Neurosci..

[42] Bergman O., Ben-Shachar D. (2016). Mitochondrial Oxidative Phosphorylation System (OXPHOS) Deficits in Schizophrenia: Possible Interactions with Cellular Processes. Can. J. Psychiatry.

[43] Ben-Shachar D. (2017). Mitochondrial multifaceted dysfunction in schizophrenia; complex I as a possible pathological target. Schizophr. Res..

[44] Kumar P., Efstathopoulos P., Millischer V., Olsson E., Wei Y.B., Brüstle O., Schalling M., Villaescusa J.C., Ösby U., Lavebratt C. (2018). Mitochondrial DNA copy number is associated with psychosis severity and antipsychotic treatment. Sci. Rep..

[45] Ermakov E.A., Dimitrieva E.M., Parshukova D.A., Kazantseva D.V., Vasilieva A.R., Smirnova L.P. (2021). Oxidative Stress-Related Mechanisms in Schizophrenia Pathogenesis and New Treatment Perspectives. Oxid. Med. Cell. Longev..

[46] Kim H.J., Hwang I.W., Kwon B.N., Han S.H., Lee N.R., Lim M.H., Kwon H.J., Jin H.J. (2019). Assessment of associations between mitochondrial DNA haplogroups and attention deficit and hyperactivity disorder in Korean children. Mitochondrion.

[47] Kim J.L., Lee S.Y., Park M., Kim J.W., Kim S.A., Kim B.N. (2019). Peripheral Mitochondrial DNA Copy Number is Increased in Korean Attention-Deficit Hyperactivity Disorder Patients. Front. Psychiatry.

[48] Bergersen L.H. (2015). Lactate transport and signaling in the brain: Potential therapeutic targets and roles in body-brain interaction. J. Cereb. Blood Flow Metab..

[49] Brooks G.A. (2009). Cell-cell and intracellular lactate shuttles. J. Physiol..

[50] Dogan A.F., Yuksel C., Chouinard V.A., Öngür D. (2018). Brain lactate and pH in schizophrenia and bipolar disorder: A systematic review of findings from magnetic resonance studies. Neuropsychopharmacology.

[51] Robinet C., Pellerin L. (2011). Brain-derived neurotrophic factor enhances the hippocampal expression of key postsynaptic proteins in vivo including the monocarboxylate transporter MCT2. Neuroscience.

[52] Suzuki A. (2011). Astrocyte-neuron lactate transport is required for long-term memory formation. Cell.

[53] Sullivan C.R., Mielnik C.A., Funk A., O’Donovan S.M., Bentea E., Pletnikov M., Ramsey A.J., Wen Z., Rowland L.M., McCullumsmith R.E. (2019). Measurement of lactate levels in postmortem brain, iPSCs, and animal models of schizophrenia. Sci. Rep..

[54] Herbeth M., Koethe D., Bahn S., Cheng T.M., Krzyszton N.D., Schoeffmann S., Guest P.C., Rahmoune H., Harris L.W., Kranaster L. (2011). Impaired glycolytic response in peripheral blood mononuclear cells of first-onset antipsychotic-naive schizophrenia patients. Mol. Psychiatry.

[55] Ľuptak M., Fišar Z., Hroudova J. (2021). Effect of novel antipsychotics on energy metabolism—In vitro study in pig brain mitochondria. Mol. Neurobiol..

[56] Bar-Yosef T., Hussein W., Yitzhaki O., Damri O., Givon L., Marom C., Gurman V., Levine J., Bersudsky Y., Agam G. (2020). Mitochondrial function parameters as a tool for tailored drug treatment of an individual with psychosis: A proof of concept study. Sci. Rep..

[57] Herbst E.A., Paglialunga S., Gerling C., Whitfield J., Mukai K., Chabowski A., Heigenhauser G.J., Spriet L.L., Holloway G.P. (2014). Omega-3 supplementation alters mitochondrial membrane composition and respiration kinetics in human skeletal muscle. J. Physiol..

[58] Robinson D.G., Gallego J.A., John M., Hanna L.A., Zhang J.P., Birnbaum M.L., Greenberg J., Naraine M., Peters B.D., McNamara R.K. (2019). A potential role for adjunctive omega-3 polyunsaturated fatty acids for depression and anxiety symptoms in recent onset psychosis: Results from a 16weekrandomized placebo-controlled trial for participants concurrently treated with risperidone. Schizophr. Res..

[59] Pawelczyk T., Grancow-Grabka M., Trafalska E., Szemraj J., Żurner N., Pawełczyk A. (2018). Telomerase level increase is related to n-3 polyunsaturated fatty acid efficacy in first episode schizophrenia: Secondary outcome analysis of the OFFER randomised clinical trial. Prog. Neuro-Psychopharmacol. Biol. Psychiatry.

[60] Pawelczyk T., Piatkowska-Janko E., Bogorodzki P., Gębski P., Grancow-Grabka M., Trafalska E., Żurner N., Pawełczyk A. (2018). Omega-3 fatty acid supplementation may prevent loss of gray matter thickness in the left parieto-occipital cortex in first episode schizophrenia: A secondary outcome analysis of the OFFER randomised controlled study. Schizophr. Res..

[61] Sepehrmanesh Z., Heidary M., Akasheh N., Akbari H., Heidary M. (2018). Therapeutic effect of adjunctive N-acetyl cysteine (NAC) on symptoms of chronic schizophrenia: A double-blind, randomised clinical trial. Prog. Neuro-Psychopharmacol. Biol. Psychiatry.

[62] Hu M., Zhang Y., Ma S., Li J., Wang X., Liang M., Sferruzzi-Perri A.N., Wu X., Ma H., Brännström M. (2021). Suppression of uterine and placental ferroptosis by N-acetylcysteine in a rat model of polycystic ovary syndrome. Mol. Hum. Reprod..

